# Magnitude of maternal near misses and the role of delays in Ethiopia: a hospital based cross-sectional study

**DOI:** 10.1186/s13104-019-4628-y

**Published:** 2019-09-18

**Authors:** Mulugeta Dile Worke, Habtamu Demelash Enyew, Maru Mekie Dagnew

**Affiliations:** 1 Department of Midwifery, College of Health Sciences, Debre Tabor University, Debre Tabor, Ethiopia; 2 Department of Public Health, College of Health Sciences, Debre Tabor University, Debre Tabor, Ethiopia

**Keywords:** Magnitude, Maternal near miss, Delays, Amhara, Ethiopia

## Abstract

**Objectives:**

This study was aimed to assess the magnitude of maternal near misses and the role of delays including other risk factors. A Hospital based cross sectional study was conducted at three referral hospitals of Amhara region on 572 mothers who came to obtain obstetrics care services from February 01 to July 30, 2018.

**Results:**

The magnitude of maternal near miss was 26.6% (95% CI 23, 30). With regards to delays, 83 (14.5%), 226 (39.5%), and 154 (26.9%) of women delayed in the decision to seek care, in reaching care, and in receiving care respectively. Women who had no antenatal care [AOR = 3.16; CI (1.96, 5.10)], who stayed in hospital 7 days or more [AOR = 2.20; CI (1.33, 3.63)] and those who had delay in reaching health facility [AOR = 1.99; CI (1.10, 3.61)] were more likely to be near miss. While, women whose husband was able to read and write [AOR = 0.29; CI (0.09, 0.96)] and those with monthly household income between 2001 and 3000 ETB [AOR = 0.35; CI (0.18, 0.70)] were 71% and 65% less likely to be near misses respectively. Promoting antenatal care and increasing maternal health care access could have significant impact in reducing maternal near misses.

## Introduction

Maternal near miss is defined as a severe life threatening obstetric complication necessitating an urgent medical interventions which leads to death if treatment is not initiated timely [1, 2]. Women who survive life-threatening conditions arising from complications related to pregnancy and childbirth have many common aspects with those who die of such complications [3–6]. Although maternal mortality remains a significant public health problem, maternal deaths are rare in absolute number due to advances medicine that make assessment of program effectiveness difficult solely by mortality intensifying the significance near miss [7]. Exploring clients who survived from life-threatening conditions provide a more complete assessment of quality of care. Near miss provides indirect evidence related to health system, emergency obstetrics services, and the standard of care [3–5].

The prevalence of maternal near-miss is high particularly in developing countries [8]. A systematic reviews on maternal near misses reported an incidence of 0.01–8.23% in studies which use different criteria [9]. A retrospective cross sectional study carried out at Ayder Hospital, Ethiopia reported a proportion of maternal near misses of 10.1% [10].

Near miss is a multifactorial condition which is caused by socio-economic, health event, skill of health care providers, and sub-standardized services [11–13]. Studies indicated that low socioeconomic status, unemployment, high parity, being single, and prior caesarean section are predictors of maternal near misses [14, 15]. The three delays are the main cause for maternal near miss [16, 17]. The first delay is related to delay in seeking care due to cultural or low awareness. The second delay is related to delay in reaching health facility due to lack of access, transport problem, and cost related issues. While, the third delay is delay in receiving care due to either lack of trained personnel, equipments or poor quality of care [15].

A multicenter cross-sectional study conducted in Brazil revealed that 68.7% of near misses were associated with delays [17]. Another study in Gabon reported that 40% of women delayed more than 45 min before seeing any qualified personnel [18]. Having nearby health facility and access to transport improves utilization of maternal health services which could reduce near misses related to the delays [19]. Hence, this study was aimed to identify the magnitude of maternal near miss and the role of delays which provide insights for stakeholders in decision making and policy development.

## Main text

### Methods

#### Setting

A hospital based cross sectional study was conducted in maternity units of selected referral hospitals in Amhara region from February 1 to July 30, 2018. The region has a total of 5 referrals hospitals. Three of the five (i.e. Felege Hiwot, Debre Markos and Debre Birhan) referral hospitals were randomly selected using lottery method. These referral hospitals render services for about 5000, 3700 and 3500 mothers per year, for Felege Hiwot, Debre Markos, and Debre Birhan respectively [20].

#### Participants

All women who were seeking maternal health care services in Amhara regional state referral hospitals were source population. While, all women who came to the selected referral Hospitals to obtain maternal health care services during antenatal or intra-partum or within 42 days after delivery were the study population.

#### Inclusion and exclusion criteria

Women who were pregnant, in labour, had abortion or who delivered in the last 42 days visiting health facilities were eligible. While, women who developed conditions unrelated to pregnancy were excluded.

#### Sampling technique and procedure

A single population proportion formula with prevalence of near miss (P) = 23.3% from previous study [21], 5% of precision, 95% confidence level, adding a 5% non-response rate, and design effect of 2 giving final sample size of 572.

The calculated sample was proportionally allocated to each hospitals based on patient flow and study participants were selected through systematic random sampling technique. The sampling intervals for each referral hospital were determined by dividing the average number of mothers visiting the hospitals during the study period per allocated sample size.

#### Variables

Maternal near miss was the dependent variable and the independent variables were demographic, socioeconomic characteristics, client characteristics, and the three delays for obstetric care services.

#### Operational definition

Maternal near miss is defined as a severe life threatening obstetric complication necessitating an urgent medical intervention to prevent the likely death of the pregnant or recently delivered woman, in whom immediate survival is threatened and whom survives by chance or because of the hospital care she received [2, 22]. The case identification was done by clinical criteria related to a specific disease entity such as severe eclampsia or hemorrhage; or interventions such as admission to an intensive care unit or a procedure such as a hysterectomy or massive blood transfusion; or a method whereby organ system dysfunction such as shock or respiratory distress [6]. Delay in reaching health facility was considered if woman travel > 10 km to get health facility [23, 33].

#### Data collection and quality assurance

Data were collected by six Midwives through interview using structured questionnaire. A medical record was also reviewed to confirm obstetric history and clinical diagnosis. The questionnaire was translated into Amharic language by experts in both languages and was translated back to English by another expert to ensure consistency. To maintain the quality of data, the data collectors were closely monitored by three medical doctors and the principal investigator. Training was given for 3 days about the study. Pretest was conducted on 30 women who came for obstetrics services in Debre Tabor Hospital before the start of actual data collection and necessary modification was made.

#### Data analysis

Data were checked for completeness, coded, entered and cleaned by EPI-Info version 3.5.3 software and analyzed SPSS version 20. Bivariable logistic regression analysis was performed. P < 0.25 was used as a criterion to select candidate variables for multivariate analysis. Multivariable logistic regression analysis was performed to the predictors of near miss at 95% CI. Variables with variance inflation factor (VIF) > 10 were excluded in the multivariable analysis to control collinearity. P value < 0.05 was used to determine significance of association.

#### Ethical considerations

Ethical clearance was obtained from University of Gondar, College Health Sciences Research Ethics Committee. In addition, official letter of cooperation was submitted to the selected hospitals to obtain permission. After clarifying the study objectives, written consent was obtained from each participant. Confidentiality was also assured at all levels of the study.

### Results

#### Socio-demographic characteristics

A total of 572 study participants were included in the study with response rate of 100%. The mean age of the respondents was 27.96 ± 6 years. More than half, (53.1%) of the study participants did not attend any formal education (Table 1).

**Table 1:** Distribution of mothers by their socio-demographic characteristic in Amhara region referral hospitals, Northwest Ethiopia, February 01 to July 30, 2018

Characteristics	Frequency (n)	Percent (%)
Age (in years) (n = 572)		
< 20	76	13.3
20–30	346	60.5
31–40	140	24.5
≥ 40	10	1.7
Marital status (n = 572)		
Single	16	2.8
Married	540	94.4
Divorced	16	2.8
Religion (n = 572)		
Orthodox	512	89.5
Muslim	54	9.4
Others (protestant, catholic)	6	1.1
Occupational status (n = 572)		
Farmer	150	26.2
Housewife	268	46.9
Government employee	71	12.4
Trader	51	8.9
Unemployed	32	5.6
Educational status (n = 572)		
No formal education	304	53.1
Grade 1–6	81	14.2
Grade 7–12	111	19.4
Above grade 12	76	13.3
Average monthly income per household (n = 572)		
< 1000 Birr	135	23.6
1001–2000 Birr	186	32.5
2001–3000 Birr	150	26.2
3001–4000 Birr	54	9.4
≥ 4001 Birr	47	8.2
Husband educational status of mothers (n = 556)		
Unable to write and read	207	37.2
Able to write and read only	44	7.9
Grade 1–6	59	10.6
Grade 7–12	101	18.2
Above grade 12	145	26.1
Residence (n = 572)		
Rural	196	34.3
Urban	376	64.5
Distance traveled (n = 572)		
≤ 10 km	147	25.7
> 10 km	425	74.3

#### Obstetric characteristics

Of the total respondents, 485 (84.8%) were visiting hospitals for the first time. Four hundred fifty-six (79.7%) were from 37 to 42 weeks of pregnancy and 425 (74.3%) were referred from other health facilities. One hundred fifty-eight (27.6%) of the women stayed 7 or more days in the hospitals, and 164 (28.7%) of mothers delivered spontaneously (Table 2).

**Table 2:** Obstetric characteristics of respondents in Amhara region referral hospital, Northwest Ethiopia, February 01 to July 30, 2018

Characteristics	Frequency	Percent
ANC visits (n = 572)		
Not booked	174	30.4
Booked	398	69.6
Number of ANC visits (n = 572)		
None	174	30.4
One visit	59	10.3
2–4 visits	299	52.3
> 4 visits	40	7.0
Visit type (n = 572)		
New visits	485	84.8
Repeat visits	87	15.2
Source of referral (n = 572)		
Health institution	425	74.3
Self-referral	147	25.7
Type of current pregnancy (n = 572)		
Wanted and supported	437	76.4
Wanted but unplanned	25	4.4
Unwanted and unplanned	110	19.2
Gestational age (n = 572)		
≤ 28 weeks	55	9.6
29 to 36 weeks	53	9.3
37 to 42 weeks	456	79.7
> 42 weeks	8	1.4
Parity (n = 572)		
≤ 1 delivery	260	45.5
2 to 4 deliveries	199	34.8
≥ 5 deliveries	113	19.8
Duration of labor (n = 489)		
≤ 24 h	365	74.6
> 24 h	124	25.4
Type of interventions (n = 572)		
SVD	164	28.7
CS	163	28.5
Hysterectomy	121	21.2
Destructive deliveries	75	13.1
MVA/D and C	49	8.6
Duration of hospital stay (n = 572)		
< 7 days	414	72.4
≥ 7 days	158	27.6

#### The delays

With regards to delays, 83 (14.5%), 226 (39.5%), and 154 (26.9%) of women delayed in the decision to seek care, in reaching care, and in receiving care respectively. The most common reason for the delay to seek care was lack of information where the service is given 40 (7%) followed by lack of information which institution is going to give the service 6 (1%). Long distance 124 (36.2%), delay to reach the place of care 72 (12.4%), and lack of transport 30 (5.2%) were commonly cited factors for delay in reaching care. Whereas, delay in correct diagnosis 32 (5.6%), patient refusal for medical help 22 (3.8%), delay in definitive treatment 23 (4%), and poor monitoring 20 (3.4%) were common reasons for delay in receiving care.

#### Magnitude of maternal near misses

The overall magnitude of maternal near miss in Amhara regional state referral hospitals was 26.6% (95% CI 23%, 30%). With regards to proportion of cases, 117 (77%) of the near misses were from rural areas and the rest 35 (23%) were from urban areas. The highest proportion of maternal near miss was reported in Felege Hiwot referral hospital 92 (60.5%) followed by Debre Birhan referral hospital 36 (23.7%) and Debre Markos referral hospital 24 (15.8%).

#### Factors associated with maternal near misses

The multiple logistic regression showed that monthly income, husband’s ability to read and write, not booked, seven or more days’ hospital stay and delay in reaching the final place of care were significantly associated with maternal near miss (Table 3).

**Table 3:** Determinants of maternal near misses in Amhara region referral hospitals, Northwest Ethiopia, February 01 to July 30, 2018

Characteristics	Maternal near miss		COR (95% CI)	AOR (95% CI)
	Yes	No		
Husband educational status (556)				
Unable to write and read	52	155	1.23 (0.74, 2.05)	0.658 (0.33, 1.33)
Able to write and read only	4	40	0.37 (0.12, 1.11)	0.29 (0.09, 0.96)^b^
Primary education	21	38	2.03 (1.05, 3.95)^a^	1.305 (0.58, 2.94)
Secondary education	37	64	2.13 (1.21, 3.75)^a^	1.623 (0.79, 3.34)
College and above	31	114	1.00	1.00
ANC follow up (n = 572)				
Not booked	76	99	3.24 (2.20,4.79)^a^	3.16 (1.96, 5.10)^b^
Booked	76	321	1.00	1.00
Monthly income (n = 572)				
< 1000 Birr	49	86	1.00	1.00
1001–2000 Birr	51	135	0.66 (0.41, 1.07)	0.58 (0.32, 1.05)
2001–3000 Birr	26	124	0.37 (0.21, 0.64)^a^	0.35 (0.18, 0.70)^b^
3001–4000 Birr	15	39	0.68 (0.34, 1.35)	0.96 (0.36, 2.46)
≥ 401 Birr	11	36	0.54 (0.25, 1.15)	0.50 (0.19, 1.36)
Duration of hospital stay (n = 572)				
< 7 days	92	322	1.00	1.00
≥ 7 days	60	98	2.14 (1.44,3.18)^a^	2.20 (1.33, 3.63)^b^
Delay in reaching the final place of care (n = 572)				
Traveled ≤ 10 km	25	122	1.00	1.00
Traveled > 10 km	127	298	2.08 (1.29, 3.35)^a^	1.99 (1.10, 3.61)^b^

^a^Significant at bivariable analysis

^b^Significant at multivariable analysis

### Discussion

The magnitude of near misses among hospital deliveries was 26.6% (CI 23%, 30%) in this study. The finding is similar with the study done at regional level [21] and in Campinas, Brazil [24]. However, it is higher than studies carried out in Uganda (10.61%) [25], Nigeria (12%) [11], and Brazil (15.8%) [26]. The higher incidence in this study might be due to low awareness of mothers on maternal near miss and high patient flow in the referral hospitals due to limited referral hospitals in the region despite high population number. With regards to delays, 83 (14.5%), 226 (39.5%), and 154 (26.9%) of women delayed to seek care, in reaching care, and in receiving care respectively. The overall delay was found to be 463 (80.9%) in this study which is higher than a study conducted in Brazil [17]. The difference might be related to variation in awareness and health infrastructure.

The odds of being near miss were found to be 2 times higher among women who stayed 7 days or more in hospitals compared with counterparts [AOR = 2.20; CI (1.33, 3.63)]. The finding is consistent with a study conducted in Brazil [27]. Studies indicated that women who had long hospital stay are at higher risk of developing infections due to illness, invasiveness of some medical procedures, overcrowding and poor infection control practices [28, 29].

The odds of being near miss were found to be 3 times higher among women who had antenatal care (ANC) compared with those with no ANC [AOR = 3.16; CI (1.96, 5.10)]. The finding suggested that routine ANC has an indirect effect on maternal near miss, possibly by avoiding the delay to seek care just by increasing awareness about timely care. This finding is consistent with studies carried out in Bolivia [30] and Ayder hospital, Ethiopia [10].

With regards to delays to reach health facility, the odds of facing near miss were found to be 2 times higher among women who traveled > 10 km compared with those who traveled ≤ 10 km [AOR = 1.99; CI (1.10, 3.61)]. The finding of this study is consistent with studies carried out in Dabat, Ethiopia [31], and Sierra Leone [32]. Poor access to health institution in nearby place, lack of road infrastructure or transport forces women to travel long distance on foot leading to delays in reaching health facilities [19]. Our study suggested that there is a need to revise the access of health facilities and transportation system in the region.

Women with monthly household income of 2001–3000 Ethiopian birr were 65% less likely to be near miss cases than women whose monthly income was less than 1000 Ethiopian Birr [AOR = 0.35; CI (0.18, 0.70)]. This might be women with better household income are more likely to be educated and capability to avoid the delays. The finding of this study is consistent with a study conducted in Hossaina, Ethiopia [33].

### Conclusions and recommendation

The magnitude of maternal near miss was high. ANC, husband’s education, delay in reaching the final place of care, household income, and hospital stay were significantly associated with maternal near miss. Therefore, promoting ANC, and increasing maternal health care access could have significant impact. In addition, provision of women centered care, and investing in health promotion can reduce maternal near misses.

## Limitations

This study does have some inherent limitations. The study design makes it difficult to determine the direction of causality. Moreover, neonatal outcomes, and maternal outcomes after 42 days were not studied and we suggest to be studied in the future. To increase the quality of data chart review was used as a supplement of interview.

## Data Availability

The datasets generated during the current study are available from the corresponding author on reasonable request.

## Abbreviations

ANC: antenatal care
AOR: adjusted odds ratio
CI: confidence interval
COR: crude odds ratio
